# Intraosseous Neurofibroma and Concurrent Involvement of the Mandible, Maxilla and Orbit: Report of a Case

**DOI:** 10.5812/iranjradiol.6684

**Published:** 2012-03-25

**Authors:** Zahra Dalili, Gholamhossein Adham

**Affiliations:** 1 Department of Maxillofacial Radiology, Dental School, Guilan University of Medical Sciences, Rasht, Iran; 2 Department of Maxillofacial Surgery, Dental School, Guilan University Of Medical Sciences, Rasht, Iran

**Keywords:** Neurofibroma, S100 Proteins, Magnetic Resonance Imaging, Cone-Beam Computed Tomography

## Abstract

Neurofibroma is an autosomal dominant disorder which has major criteria such as hyperpigmentation (cafe-au lait spots), cutaneous and subcutaneous tumors and bone deformities. In this report, a case of multifocal intraosseous neurofibroma in a 16-year-old male with right facial asymmetry, multiple unerupted maxillary posterior teeth and a previous history of infratemporal and orbital neurofibroma is presented. The majority of reported cases occurred in the posterior portion of the mandible and a limited number in the maxilla. Cone beam CT (CBCT) was performed for better evaluation of the extension and form of the maxillary and mandibular lesions. This report presents a rare situation of simultaneous peripheral neurofibromatosis (NF) and multifocal intraosseous NF in the mandible, maxilla and orbits and also focuses on advanced imaging findings of bony and soft tissue neurofibroma.

## 1. Introduction 

Neurofibroma (NF) is an autosomal dominant disorder in 50 percent of cases and in the other fifty percent, there is no negative familial trait. This group has the highest mutation rate among genetic diseases [1][2].

The two major classifications are NF-1, a generalized form, and NF-2, a central form[3].The major location of NF is the posterior portion of the mandible. A few cases have been presented in the maxilla [1]. Females are involved more than males [4]. Hyperpigmentation (cafeau-lait spots) and cutaneous or subcutaneous tumors are observed in the majority of the patients [5].

More common radiographic findings of the jaw bones are enlargement of the mandibular foramen and the inferior alveolar canal, an elongated coronoid process, a deep sigmoid notch, unerupted teeth and a cystic lesion. Neurofibroma of the orbit as an isolated entity [6] and also associated with systemic involvement [7] is also reported.

More than 40 cases of solitary neurofibroma have been reported until 2006 [1]. The majority of them occurred in the posterior portion of the mandible and a limited number in the maxilla. In this article, we report a case of neurofibroma with concurrent unilateral involvement of the maxilla, mandible, orbit and infratemporal fossa. Besides, the CBCT features of this lesion in the maxilla and the mandible, magnetic resonance imaging (MRI) findings of the lesion in the infratemporal fossa and CT findings of the orbits have been discussed. This report has focused on advanced imaging findings of this lesion, their importance in the diagnosis and occurrence of the peripheral lesion accompanied by a rare form of multifocal intraosseous neurofibroma.

## 2. Case Presentation 

The patient, a 16-year-old male, was referred with right facial asymmetry and multiple unerupted maxillary posterior teeth. There was no history of pain. The palatal enlargement was observed on the right side of the maxilla. The overlying soft tissue on the palatal side of the right posterior portion of the maxilla was soft and fluctuant without gingival ulceration and inflammation. In clinical examination, several cutaneous nodular masses and hyperpigmentations were observed. Periauricular soft tissue swelling on the left side with extension to the posterior portion of the auricle and also external auditory meatus were observed. Hypoglobus and proptosis of the left eye were detected.

**Figure 1:**
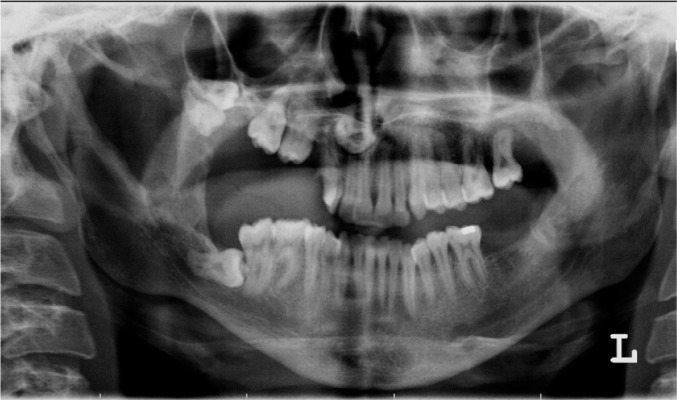
A 16-year-old male presenting with maxillary, mandibular and orbital neurofibroma. Panoramic view shows multiple tooth impactions, bone loss on the right side of the maxilla and elevation of the antral floor without a gross welldefined lesion. In the mandible, an elongated condylar neck and coronoid process, deep sigmoid notch, and oval lucency in the upper portion of the mandibular ramus are observed.

In the panoramic view, a deep sigmoid notch, an elongated left coronoid process and separated pericoronal radiolucency were found. Impaction of the right maxillary molar teeth and destruction of the overlying bone were also observed (Figure 1).

**Figure 2:**
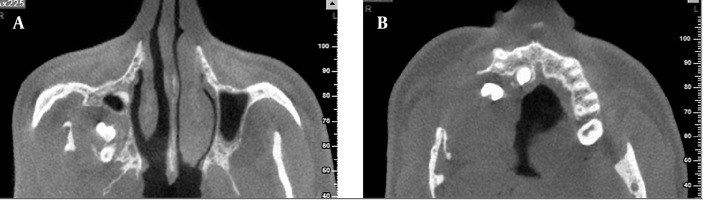
CBCT findings. A and B, Axial CBCT views indicate impacted teeth, mass effect on the posterior and lateral walls of the right maxillary sinus toward the antral cavity and involvement of the mandibular ramus.

**Figure 3:**
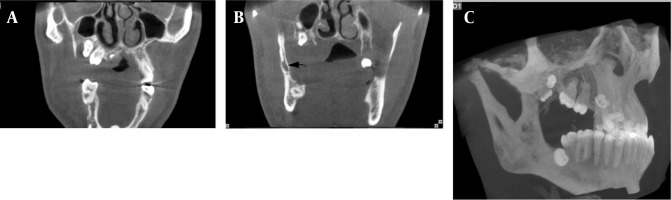
A and B, Coronal CBCT images of the same case reveal expansile mass on the right side of the maxilla with elevation of right antral floor, soft tissue bulging in palatal portion and lucent cystlike lesion in inferior portion of right ramus with thinning and expansion of cortical portion; C, 3D reconstructed image showing gross deformity of maxilla and mandible on the right side.

CBCT was carried out for better evaluation of the extension and form of the maxillary and mandibular lesions (Figure 2 and Figure 3). Axial views from the mid portion of the ramus to the level of the maxillary sinus revealed large pericoronal mass on the right side of the maxilla from the right maxillary canine to the tuberosity area with extension to the posterior portion of the right maxillary sinus and push the lateral and posterior wall of the right maxillary sinus to the antral cavity.

**Figure 4:**
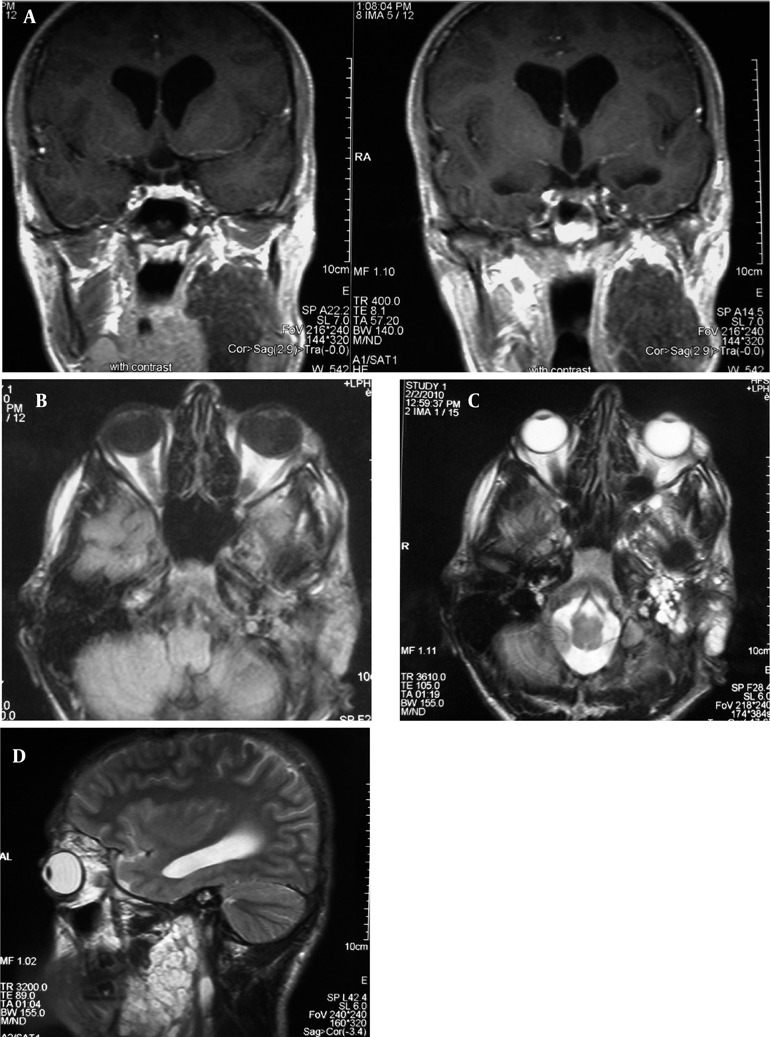
MRI findings of the infratemporal mass A, Coronal T1 weighted MRI (TR/TE = 400/8.1) showing well-defined large mass with homogeneous low signal intensity and mild internal enhancement. Pressure effect on lateral wall of naso- and oropharynx is observed; B, Axial T1 weighted image showing low signal mass adjacent to parotid gland; C and D, Axial and sagittal T2 weighted images (TR/TE = 3610/105) reveal high signal intensity of the mass in the infratemporal fossa and adjacent to the parotid gland.

Deformity of the zygomatic bone and lateral bowing and thinning of the ramus were observed. Due to preauricular soft tissue swelling and a previous history of soft tissue mass in the left infratemporal fossa, MRI was performed for re-evaluation of the left infratemporal fossa. In MRI, a huge sized tumor was seen adjacent to the parotid gland which involved the infratemporal fossa (Figure 4).

**Figure 5:**
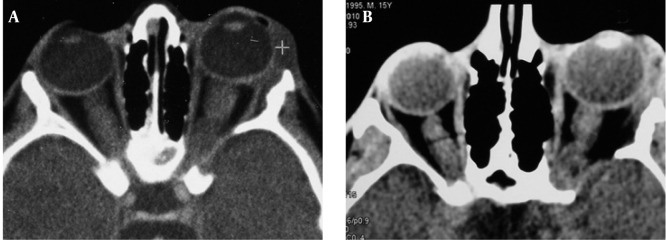
A, CT scans of the orbit at the age of 3 years and; B, The age of 15 years show soft tissue density in supralateral portion of left orbit adjacent to the globe. Thick optic nerve and bony defect in the left posterior and lateral wall of the orbit are observed.

In the evaluation of the patient’s past medical history, we found that he was a known case of intraorbital neurofibroma when he was 3 years old. Multislice CT without contrast enhancement revealed proptosis of the left eye, thick optic nerve, preseptal soft tissue density with swelling of the upper and lower eyelids and bony defect of the greater wing of the sphenoid bone (Figure 5).

**Figure 6:**
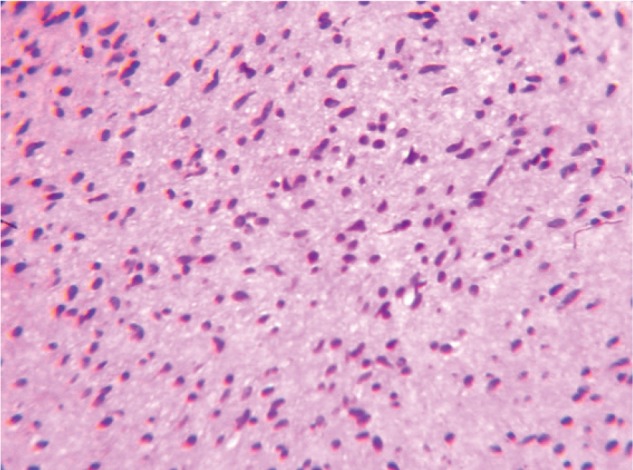
Photomicrograph shows spindle and ovoid cells in the fairly loose stroma. Wavy nuclei are observed in some of them (Hematoxyline and Eosin: magnification × 400).

Incisional biopsy of the maxillary lesion was performed. In the histopathological examination, a neoplastic structure was found in the connective tissue of the gingival mucosa, composed of spindle and ovoid cells in the fairly loose stroma and some of them had wavy nuclei (Figure 6). Methylene blue staining revealed numerous mast cells, especially near to the vessels. Histopathological findings and positive reaction for S100 protein confirmed the diagnosis of neurofibroma.

## 3. Discussion 

Intraosseous neurofibroma is relatively rare in the head and neck, with the most common site being in the mandible. Neurofibroma has been reported infrequently in the maxilla. In this case, involvement of the maxilla accompanying the mandible is the prominent point. The higher occurrence of this lesion in the mandible may be related to the long thick bundle of the inferior alveolar nerve [1]. In addition to the major criteria, axillary freckles, iris hamartoma, bone dysplasia; nervous system tumor (shwannoma) must be considered as the minor criteria [8]. Systemic involvements such as involvement of the cardiovascular, gastrointestinal and central nervous systems have been explained in the literature [9].

In the mandible, neurofibroma is accompanied with fusiform enlargement of the foramen and the inferior canal, a branched mandibular canal, a deep sigmoid notch, a decreased mandibular angle, a deformed condyle, non-eruption of the teeth and a cyst like lesion [10][11][12][13]. The stated findings were found in our case. Hypo- or hyperplasia of the mandible, maxilla, zygoma and the temporomandibular joints were also reported in these patients [1]. We also detected these findings in this case.

In the differential diagnosis of neurofibroma, shwannoma, hemangioma, lymphangioma and rhabdomyosarcoma should be considered. Orbital neurofibroma must be discriminated from optic sheath meningioma, orbital pseudotumor and granulomatous disease [14].

The simultaneous occurrence of neurofibroma and in tratumor bleeding may happen which is the result of vessel hyperplasia and aneurysm [15][16].

Low platelet sensitivity to collagen fibers in cases with neurofibroma is the rational explanation for intratumor hemorrhage and excessive bleeding during surgery [12][16][17][18]. Neurological deficits and hemorrhage are the most common complications of surgery which have been encountered [18][19].

Totally, concurrent involvement of the mandible, maxilla and orbit is not common. In this case, CBCT is required to evaluate the exact extension of the maxillary lesion and the pattern of tooth impaction in the lesion site. As a result of extension of this lesion to the posterior portion of the maxilla, exact evaluation of the pterygomaxillary fissure in order to rule out its involvement is necessary.
